# Supplementary material for: Effectiveness of digital health exercise interventions on muscle function and physical performance in older adults with possible, confirmed or severe sarcopenia: a protocol for a systematic review
Source: BMJ Open. 2024 Oct 21;14(10):e086124. doi: 10.1136/bmjopen-2024-086124 (PMC11499845; doi:10.1136/bmjopen-2024-086124)
Supplement: online supplemental file 1 [file bmjopen-14-10-s001.docx]

Flowchart 1 PRISMA-P flowchart of study selection process

**Screening**

**Eligibility**

**Identification**

**Included**

Duplicates removed (n=?)

Duplicates removed (n=?)

Not human study (n=?)

Without targeted population/intervention/ study design (n=?)

Other (i.e. conference/comment) (n=?)

Duplicates removed (n=?)

Not human study (n=?)

Unable to obtain full-text (n=?)

Not meet inclusion criteria (n=?)

Other (i.e. conference/comment) (n=?)

Total records for review

(n=?)

Randomised controlled Trial (n=?)

Cluster randomised controlled Trial (n=?)

Quasi-experimental study (n=?)

Mixed-methods study (n=?)

etc.

Records identified through electronic database searching

(n=?)

Additional records identified through other sources (e.g. snowball, web search, search verification)

(n=?)

Total records identified (n=?)

Unique records screened for title, abstract and keywords (n=?)

Full-text articles assessed for eligibility

(n=?)

Reference lists screening (n=?)

Flowchart 1 PRISMA-P flowchart of study selection process
