# Supplementary material for: Effectiveness of digital health exercise interventions on muscle function and physical performance in older adults with possible, confirmed or severe sarcopenia: a protocol for a systematic review
Source: BMJ Open. 2024 Oct 21;14(10):e086124. doi: 10.1136/bmjopen-2024-086124 (PMC11499845; doi:10.1136/bmjopen-2024-086124)
Supplement: Supplementary file 2 [file bmjopen-2024-086124.reviewer_comments.pdf]

# PEER REVIEW HISTORY

BMJ Open publishes all reviews undertaken for accepted manuscripts. Reviewers are asked to complete a checklist review form and are provided with free text boxes to elaborate on their assessment. These free text comments are reproduced below.

**ARTICLE DETAILS**

**Title (Provisional)**

Effectiveness of digital health exercise interventions on muscle function and physical performance in older adults with possible, confirmed, or severe sarcopenia: A protocol for a systematic review

**Authors**

Shi, Ya; Stanmore, Emma; McGarrigle, Lisa; Todd, Chris

---

**VERSION 1 - REVIEW**

---

| | |
|---|---|
| **Reviewer** | **1** |
| **Name** | **Chan, Karly O. W.** |
| **Affiliation** | **The Hong Kong Polytechnic University, Division of Science, Engineering and Health Studies, College of Professional and Continuing Education** |
| **Date** | **02-May-2024** |
| **COI** | **N.A.** |

---

The protocol has been meticulously prepared in accordance with the established procedures outlined in the PRISMA-P checklist.

The search strategy presented in Table 1 (p9) does not align with the second research question: 'What is the comparative efficacy of teleexercise in preventing sarcopenia, as opposed to tele-nutrition, tele-exercise and telenutrition combined, or conventional intervention?' Table 1 does not include any keywords related to nutrition. The authors should provide an explanation of how the search strategy addresses the second research question.

On page 9, line 34: Please provide further clarification on the rationale for not imposing limitations on language and publication period. What would be the course of action if an article is discovered in a language that the authors do not understand?

---

| | |
|---|---|
| **Reviewer** | **2** |
| **Name** | **Patel, Kushang** |
| **Affiliation** | **University of Washington** |
| **Date** | **21-Jun-2024** |
| **COI** | **I have no competing interests to disclose.** |

---

1. The title and introduction section indicate that this systematic review will examine tele-exercise for prevention of sarcopenia, but the eligibility criteria for trials includes participants who already have sarcopenia or reduced muscle strength or mass. Consider specifying that this systematic review will examine tele-exercise as a treatment for sarcopenia or tertiary prevention strategy.

2. Related to the previous comment, a weakness that should be considered is that by focusing on tele-exercise trials of sarcopenia, this systematic review might misestimate the effects of tele-exercise on muscle strength and physical performance outcomes among older adults as there are tele-exercise trials reporting these outcomes but in different clinical populations that also likely have muscle weakness (e.g., older adults with chronic musculoskeletal pain).

3. A major potential benefit of tele-exercise is expanding access to hard-to-reach populations, such as older adults who are homebound or who live in rural areas. Consider incorporating this as part of the rationale for the systematic review.

4. The eligibility criteria for the interventions (beginning on page 8, line 58 to page 9, line 6) were somewhat vague. Other than specifying >=4 weeks of exercise, more detail on the types, frequency, and intensity of exercise would be helpful.

5. For physical performance outcomes (lines 31-34 on page 9), self-reported measures of disability and physical activity were specified as examples, but physical performance is typically assessed using performance-based outcome measures.

6. For intervention characteristics, consider extracting mode of delivery (synchronous/asynchronous) and types of platforms and devices used.

7. The Introduction highlights a higher prevalence of sarcopenia in Asia, but this might be due to differences in case definitions or sampling. It would be useful to specify which definitions of sarcopenia were used in the prevalence studies conducted in Asia (lines 10-20, page 6).

---

## VERSION 1 - AUTHOR RESPONSE

Dear Editor and Reviewers:

Manuscript ID number: bmjopen-2024-086124

Title: Effectiveness of tele-exercise on muscle function and physical performance in older adults for preventing sarcopenia: A protocol for systematic review

Thank you so much for your insightful feedback on our manuscript. We have revised and responded to each comment in accordance with your suggestion, as demonstrated below.

EDITOR'S COMMENTS

1.Please complete a thorough proofread of the text and correct any spelling and grammar errors that you identify.

We have read through the whole manuscript and corrected the spelling and grammar errors.

2.Please consider revising the title to more accurately reflect the research question in relation to the focus on prevention and/or treatment of sarcopenia.

Thank you so much for your suggestion. We have changed the title to " Effectiveness of digital health exercise interventions on muscle function and physical performance in older adults with possible, confirmed, or severe sarcopenia: A protocol for a systematic review".

According to the European Working Group on Sarcopenia in Older People and the Asian Working Group for Sarcopenia, sarcopenia can be classified into three categories: possible; confirmed; and severe sarcopenia. Possible sarcopenia is the early stage of confirmed sarcopenia. We will select studies including participants with the three conditions. Digital health exercise intervention in this paper is seen as a treatment for sarcopenia but also a primary prevention strategy. (P1, P3)

3.Formatting Amendments (where applicable)

We have checked the format of the article and adjusted it according to the journal's requirements.

REVIEWER COMMENTS

Reviewer: 1

Dr. Karly O. W. Chan, The Hong Kong Polytechnic University

Comments to the Author:

1.The protocol has been meticulously prepared in accordance with the established procedures outlined in the PRISMA-P checklist.

Thank you very much for your comments, we have revised the article according to your advice.

2.The search strategy presented in Table 1 (p9) does not align with the second research question: 'What is the comparative efficacy of tele-exercise in preventing sarcopenia, as opposed to tele-nutrition, tele-exercise and telenutrition combined, or conventional intervention?' Table 1 does not include any keywords related to nutrition. The authors should provide an explanation of how the search strategy addresses the second research question.

Thank you so much for your valuable advice! We had also considered this and had revised our search strategy in a way that reflects your suggestions. The new strategy includes not only exercise and nutrition, but also health education. (P9-10)

3.On page 9, line 34: Please provide further clarification on the rationale for not imposing limitations on language and publication period. What would be the course of action if an article is discovered in a language that the authors do not understand?

Thanks for your comments. In order to broaden the search scope, we ultimately decided not to restrict the time and language, as we conducted a preliminary search in the early stages and discovered that the number of papers was small. In terms of language, numerous translation software is available and capable of satisfying our requirements. We will also consult with appropriate language instructors in a timely manner if we encounter material that is challenging to comprehend.

Reviewer: 2

Dr. Kushang Patel, University of Washington

Comments to the Author:

1.The title and introduction section indicate that this systematic review will examine tele-exercise for prevention of sarcopenia, but the eligibility criteria for trials includes participants who already have sarcopenia or reduced muscle strength or mass. Consider specifying that this systematic review will examine tele-exercise as a treatment for sarcopenia or tertiary prevention strategy.

Thank you so much for this comment.

Please see above the response for the second question to the editor who made the same comment. (P1, P3)

2. Related to the previous comment, a weakness that should be considered is that by focusing on tele-exercise trials of sarcopenia, this systematic review might misestimate the effects of tele-exercise on muscle strength and physical performance outcomes among older adults as there are tele-exercise trials reporting these outcomes but in different clinical populations that also likely have muscle weakness (e.g., older adults with chronic musculoskeletal pain).

Thank you so much for this comment. Based on the responses to the previous question, we decided to focus on the keyword “sarcopenia”. We will only include studies where participants clearly fulfil the sarcopenia definition (possible; confirmed; or severe sarcopenia). This avoids confusion with other muscle disorders. (P7)

We have changed our search strategy accordingly, which will make the search results more accurate. The keywords related to sarcopenia are the key indexes in the diagnosis of sarcopenia. (P9-10)

3. A major potential benefit of tele-exercise is expanding access to hard-to-reach populations, such as older adults who are homebound or who live in rural areas. Consider incorporating this as part of the rationale for the systematic review.

Thank you very much for your advice. We have added these benefits in the background section. (P6)

4. The eligibility criteria for the interventions (beginning on page 8, line 58 to page 9, line 6) were somewhat vague. Other than specifying >=4 weeks of exercise, more detail on the types, frequency, and intensity of exercise would be helpful.

We appreciate your feedback. Given that this is a systematic review, our objective is to conduct a systematic search and organization of information regarding tele-exercise for sarcopenia. Therefore, the eligibility criteria are unable to explicitly define the type, frequency, intensity, and other information about tele-exercise, as these details are crucial results during our systematic review. We have already provided a comprehensive explanation of these information in the "data extraction" section. To address any lack of clarity we will use TiDIER to guide our description of interventions identified and have added this to the protocol. (P10)

5. For physical performance outcomes (lines 31-34 on page 9), self-reported measures of disability and physical activity were specified as examples, but physical performance is typically assessed using performance-based outcome measures.

Thank you so much for your advice. We have removed such subjective indices as examples in the physical performance outcomes. (P8)

6. For intervention characteristics, consider extracting mode of delivery (synchronous/ asynchronous) and types of platforms and devices used.

Thank you so much for your suggestion. We have added these information in the "data extraction" section as you suggested and will use TiDIER to guide our description of interventions (and the ProFaNE taxonomy of exercise interventions). (P10)

7.The Introduction highlights a higher prevalence of sarcopenia in Asia, but this might be due to differences in case definitions or sampling. It would be useful to specify which definitions of sarcopenia were used in the prevalence studies conducted in Asia (lines 10-20, page 6).

Thank you so much for your comment. We have rewritten this section of the introduction to clarify prevalence rates in Europe and Asia and trust this removes any ambiguity and addresses the issue of case finding methods. (P5)

Finally, we really appreciate all the time and effort you put into this document, as well as all your insightful suggestions for revising the manuscript to make it more readable, clear, and academic. Thank you so much once more!

Best wishes

| **Reviewers comments** | **Response** |
|---|---|
| Reviewer: 1<br>Dr. Karly O. W. Chan, The Hong Kong Polytechnic University<br>Comments to the Author:<br><br>The protocol has been meticulously prepared in accordance with the established procedures outlined in the PRISMA-P checklist. | Thank you very much for your comments, we have revised the article according to your advice. |
| The search strategy presented in Table 1 (p9) does not align with the second research question: 'What is the comparative efficacy of tele-exercise in preventing sarcopenia, as opposed to tele-nutrition, tele-exercise and telenutrition combined, or conventional intervention?' Table 1 does not include any keywords related to nutrition. The authors should provide an explanation of how the search strategy addresses the second research question. | Thank you so much for your valuable advice! We had also considered this and had revised our search strategy in a way that reflects your suggestions. The new strategy includes not only exercise and nutrition, but also health education. |
| On page 9, line 34: Please provide further clarification on the rationale for not imposing limitations on language and publication period. What would be the course of action if an article is discovered in a language that the authors do not understand? | Thanks for your comments. In order to broaden the search scope, we ultimately decided not to restrict the time and language, as we conducted a preliminary search in the early stages and discovered that the number of papers was small. In terms of language, numerous translation software is available and capable of satisfying our requirements. We will also consult with appropriate language instructors in a timely manner if we encounter material that is challenging to comprehend. |
| Reviewer: 2<br>Dr. Kushang Patel, University of Washington<br>Comments to the Author:<br><br>1. The title and introduction section indicate that this | Thank you so much for this comment.<br>Please see above the response for the second question to the editor who made the same comment. |

| | |
|---|---|
| systematic review will examine tele-exercise for prevention of sarcopenia, but the eligibility criteria for trials includes participants who already have sarcopenia or reduced muscle strength or mass. Consider specifying that this systematic review will examine tele-exercise as a treatment for sarcopenia or tertiary prevention strategy. | |
| 2. Related to the previous comment, a weakness that should be considered is that by focusing on tele-exercise trials of sarcopenia, this systematic review might misestimate the effects of tele-exercise on muscle strength and physical performance outcomes among older adults as there are tele-exercise trials reporting these outcomes but in different clinical populations that also likely have muscle weakness (e.g., older adults with chronic musculoskeletal pain). | Thank you so much for this comment. Based on the responses to the previous question, we decided to focus on the keyword "sarcopenia". We will only include studies where participants clearly fulfil the sarcopenia definition (possible; confirmed; or severe sarcopenia). This avoids confusion with other muscle disorders.<br><br>We have changed our search strategy accordingly, which will make the search results more accurate. The keywords related to sarcopenia are the key indexes in the diagnosis of sarcopenia. |
| 3. A major potential benefit of tele-exercise is expanding access to hard-to-reach populations, such as older adults who are homebound or who live in rural areas. Consider incorporating this as part of the rationale for the systematic review. | Thank you very much for your advice. We have added these benefits in the background section. |
| 4. The eligibility criteria for the interventions (beginning on page 8, line 58 to page 9, line 6) were somewhat vague. Other than specifying >=4 weeks of exercise, more detail on the types, frequency, and intensity of exercise would be helpful. | We appreciate your feedback. Given that this is a systematic review, our objective is to conduct a systematic search and organization of information regarding tele-exercise for sarcopenia. Therefore, the eligibility criteria are unable to explicitly define the type, frequency, intensity, and other information about tele-exercise, as these details are crucial results during our systematic review. We have already provided a comprehensive explanation of these information in the "data extraction" section. To address any lack of clarity we will use TiDIER to guide our description of interventions identified and have added this to the protocol. |
| 5. For physical performance outcomes (lines 31-34 on page 9), self-reported measures of disability and physical activity were specified as examples, but physical performance is typically assessed using performance-based outcome measures. | Thank you so much for your advice. We have removed such subjective indices as examples in the physical performance outcomes. |
| 6. For intervention characteristics, consider extracting mode of delivery (synchronous/asynchronous) and types of platforms and devices used. | Thank you so much for your suggestion. We have added these information in the "data extraction" section as you suggested and will use TiDIER to guide our description of interventions (and the ProFaNE taxonomy of exercise interventions). |
| 7. The Introduction highlights a higher prevalence of sarcopenia in Asia, but this might be due to differences in case definitions or sampling. It would be useful to specify which definitions of sarcopenia were used in the prevalence studies conducted in Asia (lines 10-20, page 6). | Thank you so much for your comment. We have rewritten this section of the introduction to clarify prevalence rates in Europe and Asia and trust this removes any ambiguity and addresses the issue of case finding methods. |
